# Explanation of the Professional Development Process of General Surgery Residents in the Operating Rooms: A Situational Analysis

**DOI:** 10.30476/JAMP.2022.91510.1448

**Published:** 2022-07-01

**Authors:** LEILA SADATI, SHAHRAM YAZDANI, BABACK SABET, PEIGHAM HEIDARPOOR

**Affiliations:** 1 Department of Operating Room, School of Allied Medical Sciences, Alborz University of Medical Sciences, Karaj, Iran; 2 Department of Medical Education, Virtual School of Medical Education & Management, Shahid Beheshti University of Medical Sciences, Tehran, Iran; 3 Faculty of Medicine, Shahid Beheshti University of Medical Sciences, Tehran, Iran

**Keywords:** Socialization, Residency, Surgery, Education

## Abstract

**Introduction:**

Numerous factors and elements are effective in the professional development of any field of study, including the educational structure, the individual characteristics of learners, and the educational atmosphere prevalent in the educational environment. Understanding each of these factors and elements and the relationships among them can guide educational system administrators in the direction of professional development. Surgical residents’ professional development is no exception to this rule. As a consequence, the present research sought to explain and suggest a model for surgical assistant professional growth in Iranian operating rooms.

**Methods:**

The present research was a grounded theory study based on a post-positivist approach, in which data analysis was performed using Clark’s situational analysis methodology by drawing three maps, situational map, social worlds/arenas map, and positional map.

**Results:**

In the presence of human and non-human factors, cultural, political, historical, and social components, the ordered situational map demonstrated the complexity of the operating room learning environment. The social worlds/arenas map confirmed the existence of several communities of practice wherein surgical residents were present with different power roles, and the positional map showed role of the educational level in the acquisition of the competence in the professional development pathway. Finally, the Triple Helix model of professional development was extracted, which has three components: psychological identity, social identity, and surgical competency.

**Conclusion:**

The surgical residents’ professional development in operating rooms occurs due to the acquisition of surgical competency along with the growth of individuals and socialization. As a result, all factors and components impacting the residents' competence development process in this learning environment must be identified and their linkages clarified.

## Introduction

Despite the emphasis on competency-based education in contemporary medical education, studies indicate that some surgical residents, despite passing the residency and earning high marks on periodic evaluations and specialized board tests, do not graduate with sufficient professional competency ( 1 ). Kagbil (2016) argues that some recent graduates are afraid to do surgery independently in terms of a lack of necessary professional competency and require postgraduate training to become expert surgeons ( 2 ). George et al. (2018) investigated 536 surgical residents’ preparation for autonomous work and rated their performance after observing 10,000 and 1130 operations performed by 444 licensed surgeons. Residents lacked the capacity to perform some major surgeries autonomously, according to the findings, necessitating a remedial program to increase expertise ( 3 ).As a result, worries about residents’ independence, acquiring appropriate competency, and preparing them for safe and independent work appear to be growing, as does the need for structural reforms in order to create a competency-based education system ( 4 , 5 ). Therefore, it seems essential to revise the curriculum and place a premium on adopting competency-based teaching methods and structured evaluation ( 6 ). Several theories, including the situated learning theory, argue against the efficacy of the teacher-student teaching method and describe how learning occurs through active engagement in a community of practice and socialization process ( 7 , 8 ). Furthermore, Richard et al. (2018) stated that communities of practice could serve as a helpful guide to determine the effectiveness of medical education and assist learners and educators in coping with the complexities of medical education ( 9 ), implying that the role of these communities in training surgical assistants should be considered.

Individuals learn a series of activities in a community of practice via the well-known socialization process, and they internalize the norms and values governing that group ( 10 ). Thus, the individual identity grows and evolves in the midst of this community of practice. In disciplines such as general surgery, where learning occurs via the active engagement in a surgical team and the presence of a diverse range of people in various roles, the community of practice plays an important role in education, the formation of individual and social identity, and professional development ( 11 ). During their training, surgical residents join a variety of communities of practice, including a defined professional identity created during the general medicine course. Their shift from general medicine to residency, on the other hand, exposes them to a new social milieu, new clinical experiences, and a new curriculum, all of which might affect the process of building their individual and social identities in a new community of practice ( 12 , 13 ). According to studies, accepting newcomers into communities of practice requires them to alter their past identities and acquire a new one which can be extremely difficult. This is accomplished via the repeated appraisals of the former identity in a dynamic process and socialization ( 3 ). By recognizing the fundamentals and principles of this theory, the surgical instructors can facilitate constructive interactions within these communities, enhance the learning environment, and provide learners with additional learning opportunities, thereby assisting in forming a new professional identity and enhancing the professional competency of surgical residents ( 11 ). According to Yazdani et al. (2020), professional competency serves as the connection between the psychological and social components of professional identity, providing the learner with the necessary self-confidence to perform a surgery. On the other hand, acceptance in the community of practice allows the learner to turn from an observer into a supervisor ( 14 ) and take a step toward professional development. Undoubtedly, the professional development is not a linear process, and gaining competency, and increasing confidence to achieve professional development occur cyclically and intermittently and under the influence of various factors ( 15 ), including the individual characteristics, educational climate, and educational system governing the discipline. Considering the complexities of operating room learning environment and the importance of training surgical residents in this learning environment, the researchers in the present study decided to identify the elements and components related to the professional development of surgical residents using the situational analysis method and draw their relationships in the direction of professional development of surgical residents and present them in the form of a professional development process model for general surgical residents in Iran.

## Methods

The present study was a grounded theory study based on a post-positivist and constructivist approach in which data analysis was performed using Clark’s situational analysis methodology. Clark’s approach was used because the surgical residents are trained in the operating room as a complex environment in terms of the physical structure with various and complex interactions between human and non-human factors. Clark recommends the situational analysis methodology to identify and analyze the interactions and actions which govern the system of such features ( 16 ).

### Research setting and participants

The research setting included the operating rooms of eight teaching hospitals in Iran. In the beginning, the study samples were selected through convenience sampling. Then, based on inclusion criteria, participants with the following properties entered to study. Sampling proceeded based on theoretical sampling for distinguishing human and non-human features and building relationship maps throughout the analysis process. 

### Data collection method

Data were collected in the study via 26 semi-structured interviews and 82 hours of observation in the operating room. The interviews lasted 25 to 45 minutes. Interviews and observations were transcribed. Questions in interviews included: Can you describe your workday training program? Please tell me about your training program in these few years (teaching methods, assessment, and grading)? what, how, and from whom did you learn? What is the difference between your training and other residents outside the operating room? How and from whom did you learn the basic principles of aseptic and sterilization, tools, and equipment? How and for how long were you able to find your desired and influential position in the surgery team? What abilities do you think you have gained over the years? Do you still have weakness? Why? How would you describe a good and capable surgeon? In your opinion, what skills should be acquired to become a good and capable surgeon?

Moreover, the examples of questions that observation was done based on it, were • What is the interaction situation like? • When and how does interaction occur? • What is happening? • What are the main and silent actors (human and non-human) in the operating room environment? • Does being responsible affect the performance of the main actors? • How can actors become members of a group and maintain their membership? • What capabilities do environmental actors use? • What motivations and techniques do people use to participate and learning? • What goals do people pursue? • What rewards do people who try to be better in the group get? • What are the consequences for those who fail to enter and maintain a position in the group?

### Data analysis

**Figure 1:**
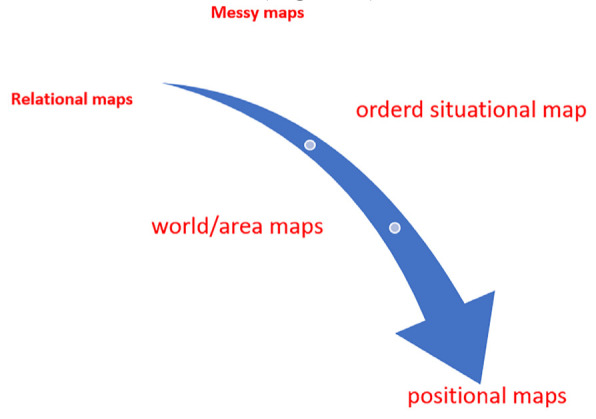
Study pathway

According to Clark’s situational analysis, data analysis was performed by drawing three maps, namely situational maps, social worlds/arenas, and positional maps. The mapping of conceptual relationships is the most advanced method in Clark’s work ( 17 ). The analysis process began with the implementation of interviews and observations transcribed on paper; the interviews were read line by line, and the initial open codes were extracted from them. The extract codes were recorded on large maps. Field notes, documents, and the memo, key and valuable topics in Clark’s situational analysis process, were also conducted simultaneously with the analysis process. After drawing many messy maps, as the first map of Clark’s maps, the specified elements were classified within a particular framework, and the ordered situational map was drawn. The researchers continued their analysis and drew worlds/arenas maps by identifying different communities of practice in the operating room displaying the existing relationships among residents and individuals, groups, and organizations. After reviewing data in two above maps, the positional map was drawn in two axes (x) and (y), based on residency level role in his/her power in the community of practice and autonomy in teaching as a supervisor and learning and *competency acquisition*. Positional maps highlight the range of positions on issues, not those associated with individuals, groups, or institutions but positions in discourses as reflected in the data. (Figure 1)

### Validity and reliability of the study

To strengthen the data, we used Guba and Lincoln’s five criteria of credibility, transferability, dependability, authenticity, and conformability ( 18 ). Besides, long-term engagement in a subject, member checking, and the triangulation method in data collection, including interviews, observations, memos, field notes, and available documents, increased the study’s validity. To achieve the greater validity of findings, the sampling with a maximum variability (age, sex, marital status, operating rooms at various universities, and residency level) was used to make more extensive data. Undoubtedly, the author’s background in teaching and research in the field of the operating room and medical education contributed to the theoretical sensitivity and increased the validity of the study and the collected data. In addition to above issues, peer review in monthly reporting provided appropriate and significant roles in the validity of the study.

### Ethical considerations

The present paper is based on a Ph.D. thesis approved by the ethics committee of Shahid Beheshti University of Medical Sciences with the code of ethics (IR.SBMU.SME.REC.1398.057). To observe the ethical principles to conduct the research, researchers received verbal consent from all participants. Furthermore, they were ensured that the interviewees’ identities would be kept confidential, and their voice files would be deleted after the study. 

## Results

Situational map as the first map of Clark’s situational analysis showed that the professional development of surgical residents was formed based on their interactions with various human and inhuman elements including residents of different years, operating room staff, surgical fellowships, surgical professors, numerous technologies, including laparoscopy, new electrical homeostasis devices, computer data recording systems, new equipment and tools and it was influenced by several components, including junior-senior culture in operating rooms, knowledge and communication skills, and personal characteristics, hierarchical discourse and lobbying, gender discrimination, current policies, including the revenue of hospitals, economic conditions and sanctions and lack of surgical equipment, historical developments and people’s desire to perform less invasive and weight loss surgeries, social maturity and the growing awareness of the public about ensuring safety by the surgeon and complaints related to medical malpractice and concerns about giving independence.

**Figure 2:**
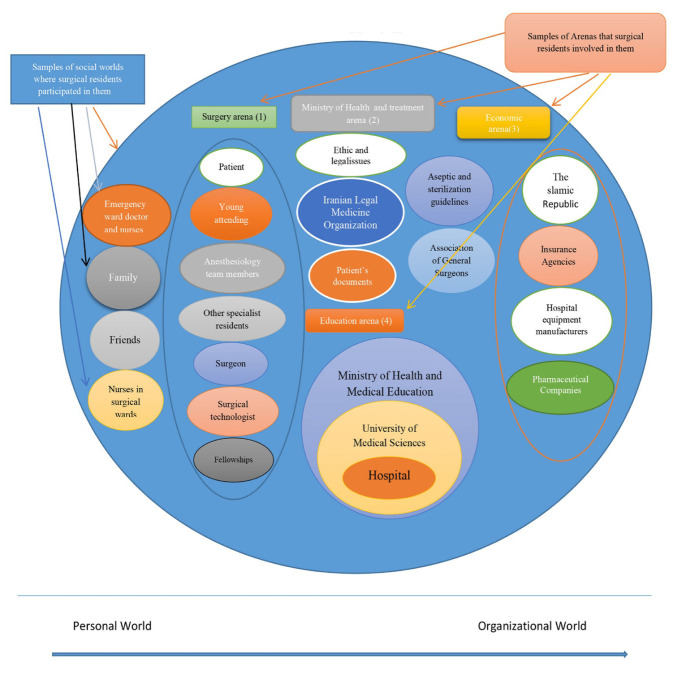
Social worlds/arenas map

Data analysis of social world/arenas maps indicated the presence of numerous arenas, including education, economics, health, and surgery, all of which surgical residents belonged to. Besides, various worlds, including the world of family interactions, friends, residents of the same year and same field, junior and senior residents, and interactions with insurance organizations were identified and recognizing each of these communities and the ability of residents to enter each of them as a community of practice play an effective role in their professional development (Figure 2).

**Figure 3:**
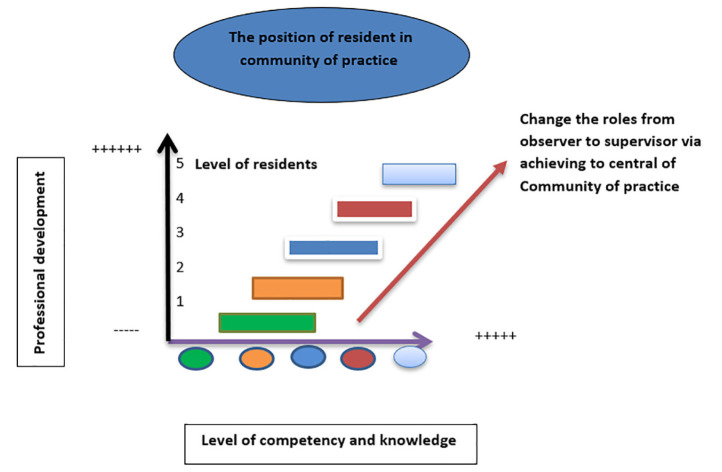
positional map

The positional map was depicted by focusing on the discourses of power and knowledge during the professional development process and by showing the residents’ situation during five years. Due to a lack of knowledge and skills in the first year, residents serve only as observers on the periphery of the surgical team’s community of practice; however, as the academic years progress, they are allocated easy procedures and thus serve as supervisors and instructor for junior residents. Over time, their surgical competency and self-confidence enable them to shift from the margins to the center of the community of practice and socialization (Figure 3).

**Figure 4:**
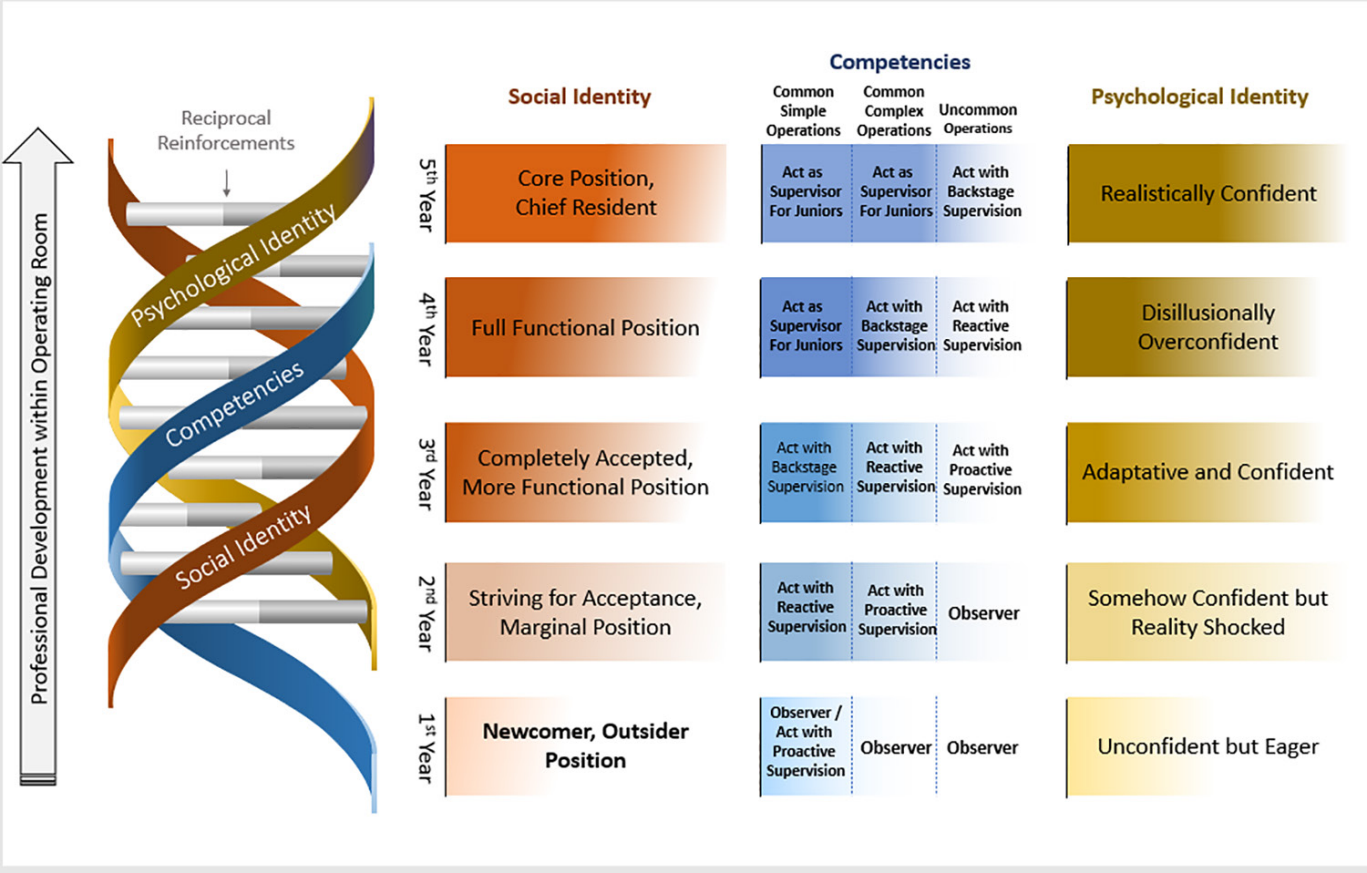
Triple Helix Model of Professional Development during Surgical Residency

At the end of analysis, it was cleared that the professional development of surgical assistants in a 5-year academic period is based on the acquisition of skills and knowledge. On the other hand, attainment of surgical skills depends on a residents' capacity to enter the surgeons' community of practice and earn the opportunity to practice under the supervision of professors. Moreover, the ability to enter this community depends on individuals' characteristics such as intelligence, communication skills, and so on. At the same time, the intelligent person finds his way to attend to the surgical team. Hence, he is given the opportunity to learn and practice. Thus, his competencies are developed, and his self-confidence are increased, so he is guided to the heart of the surgeons ‘community of practice. Over time, the professors trust him and give him the autonomy to surgery. It means that three elements, including psychological identity, social identity, and surgical competency, develop at the same time and together. Therefore, the triple helix model of professional development was drawn based on this argument (Figure 4).

## Discussion

The study’s results in terms of creating a situational map of the operating room indicated the complex interactions between various human and non-human elements, such as the multiplicity and diversity of surgical team members, the influx of new technologies, a focus on patient safety, and teamwork, all of which had an effect on the surgical residency training process. Therefore, Kopelman et al. (2013) argue that modern operating rooms are crowded with varied groups and complex technologies ( 19 ). According to Meek (2017), due to the evolution and complexity of the technologies intended for the construction of operating rooms, the interaction of non-human elements with human factors should be considered in the operating room while designing these technologies ( 20 ). Attri et al. (2015) discussed the operating room as one of the most complicated learning contexts. They believe that the concurrent presence of various surgical and anesthesia groups at the patient’s bedside, as well as their responsibilities for patient safety, necessitate significant interdisciplinary interactions and relationships ( 21 ).

On the other hand, data analysis in the situational map confirms the senior-junior relationship in the operating room and contemporary discourses, including humiliation, discrimination, and the use of lobbying to get learning opportunities and professional development. Torring et al. (2019) emphasized complex operating room relationships. They identified four distinct types of communication in the operating room: proactive communication, silent communication, ambiguous communication, and contradictory communication ( 22 ) that support the findings of current study. According to the situational map, one of the most significant human elements in the residents’ professional development was the residents themselves, who could take a step toward professional development through their individual abilities such as effective communication skills and strong basic knowledge. According to Thomas J (2018) and Locklear (2017), the professional development of a learner is a purely personal experience that depends on their ability to interact with people in a new community and to integrate previous professional identities with new ones ( 23 , 24 ).

When social worlds and arenas were mapped, various groups and arenas were found that had surgical residents as members, highlighting the role of communities of practice in the professional training and development of surgical residents. Dimitris et al. (2014) believe that in surgical education, there are several communities comprised of various kinds of residents, surgeons, and other groups, with residents having different power and governance in terms of hierarchy ( 25 ). Gandamihardja also states that, while a resident’s educational level may influence their community of practice, and a junior resident may have a different community of practice from a senior or attending resident and a surgical professor, the role of communities of practice in educating residents is well established at various levels ( 8 ). This study mentions various arenas and worlds such as health, surgery, economics, and education, as well as the world of communication with residents of the same field and level, residents of other fields, and emergency personnel which each require several individual competencies, including communication skills and arena-specific competencies, as well as acceptance of the prevailing culture of that community, and thus confirms the findings of the above studies. Gandamihardja (2019) emphasized the role models in the education of surgical residents and believed that it was necessary to establish a welcoming community in the operating room for residents, emphasize role models, implement effective mentoring, experimental learning, and reflection, and develop strategies for the joining of faculty members into a community of practice of surgeons so that residents construct their identities ( 11 ). Natan Capido (2019) also showed that residents’ attitudes and personal values change significantly within a year of entering the operating room, which changes their professional identity ( 26 ). Shahr et al. believe that professional development is formed by professional socialization in a non-linear, continuous, interactive, variable, evolutional, personal, psychosocial process. Newcomers accept it via the internalization of the specific culture of community of practice, including expectations, values, beliefs, customs, traditions, and unwritten rules of the profession, as well as understanding the hierarchy, structure of power, and responsibilities ( 27 ). This confirms Lave and Wenger’s situated learning theory that learning and acquiring skills occurs in interacting with others and by learners’ entrance to the center of the community of practice ( 28 ).

The results of the positional map indicate the shift in the situation of residents from the margins to the center of the community of practice in the vertical axis and change of their role from a mere observer to a supervisor in line with the promotion of academic years in the horizontal axis, which occurs by the acquisition of surgical competency and an increase in confidence, shifting them from a novice observer to a skilled supervisor. The findings in the vertical axis are consistent with Dreyfus and Dreyfus’s model that plots an individual’s progression via a series of five levels: novice, advanced beginner, competent, proficient, and expert ( 29 ) and in the horizontal axis, they correspond to Ten Cate’s supervision training model ( 30 ).

Finally, although Ten Cate supervision model in the horizontal axis of positional map accurately considers five levels of knowledge and skills of residents in simple and complex tasks from a mere observer to an independent supervisor, it does not explain non-technical skills, including communication skills, which play an important role in entering communities of practice. Moreover, Banner (1984) argues that the Dreyfus model is a fully developmental model based on real experience learning, which requires a learner engaged in activity rather than an expert who skillfully applies well-organized and pre-determined knowledge ( 31 ). So, since it is not a content model, the expertise components in the competency development process are not specified. Besides, it does not explain how to acquire skills, particularly complex skills through training, so this model alone cannot describe surgical residents’ process of competency development regardless of the expertise content, training method, and the complex tasks assigned to them.

Therefore, regarding the theoretical gap in the above theory and model, the spiral model of psychological identity, social identity, and surgical competency was extracted according to the analysis of data obtained from all three maps, indicating the relationship between competency and confidence of surgical assistants to enter into the community of practice of surgeons, and the shift of their roles and promotion of their social status from the legitimate peripheral to the core of this community. This shift of situation allows them to play the role of senior resident and supervisor with more confidence, perform more surgeries and gain experience by teaching others, thus paving the way for professional development. Cruess and Cruess (2014) illustrated the relationship between identity and socialization in the community of practice of medical students in a conceptual framework and mentioned the role of learning environment, education system, family, and friends in the formation of individual identity ( 32 ) Yazdani et al. (2019) believe that various factors can be effective in the socialization process, including individual factors (gender, age, race, religion, nationality, culture, personality traits, socioeconomic status, marital status, personal experiences, and motivation), organizational factors (explicit and implicit curriculum, formal and informal learning environments, system patterns and structure), and interpersonal relationships (interpersonal relationships with faculty members, staff, family, and friends) as well as feedback and reflection ( 27 ). The results of Cruess’s study (2015) confirm the effect of individual characteristics and experience in the form of reflection on the socialization and professional development of learners ( 33 ). These findings are consistent with the present study results regarding the importance of paying attention to the individual characteristics of each learner, providing an interactive and fear-free educational climate, and using structured teaching methods such as supervision models for surgical residents in achieving professional development.

### Limitation

Considering the distance and the impossibility of traveling to other cities, the researcher was only able to attend the Tehran and Karaj hospitals to collect data by observation.

## Conclusion

The results of this study showed that the professional development of surgical residents in the operating room occurred with the growth and development of psychological identity and social identity, and surgical competency which are influenced by complex interactions between human and non-human elements in the operating room. Identifying these elements, creating an interactive structured educational environment, providing educational opportunities, and deliberated practices can lead to the acquisition of surgical competency in residents and pave the way for professional development.

## Authors' contribution

L.S, Sh.Y, B.S, P.H contributed to the conception and design of the work; the acquisition, analysis, or interpretation of data for the work. All Authors contributed in drafting and revising the manuscript critically for important intellectual content. All authors have read and approved the final manuscript and agree to be accountable for all aspects of the work in ensuring that questions related to the accuracy or integrity of any part of the work are appropriately investigated and resolved.

## Conflict of Interest:

None declared.
